# Low Cerebrospinal Fluid Levels of Hemopexin Are Associated With Increased Alzheimer's Pathology, Hippocampal Hypometabolism, and Cognitive Decline

**DOI:** 10.3389/fmolb.2020.590979

**Published:** 2020-12-18

**Authors:** Azhaar A. Ashraf, Melanie Dani, Po-Wah So

**Affiliations:** ^1^Department of Neuroimaging, Institute of Psychiatry, Psychology and Neuroscience, King's College London, London, United Kingdom; ^2^Imperial College London Healthcare National Health Service Trust, London, United Kingdom

**Keywords:** Alzheimer's disease, amyloid, cerebrospinal fluid, hemoglobin subunits, hemopexin, iron, mild-cognitive impairment, tau

## Abstract

Brain iron dyshomeostasis is a feature of Alzheimer's disease. Conventionally, research has focused on non-heme iron although degradation of heme from hemoglobin subunits can generate iron to augment the redox-active iron pool. Hemopexin both detoxifies heme to maintain iron homeostasis and bolsters antioxidant capacity via catabolic products, biliverdin and carbon monoxide to combat iron-mediated lipid peroxidation. The aim of the present study was to examine the association of cerebrospinal fluid levels (CSF) hemopexin and hemoglobin subunits (α and β) to Alzheimer's pathological proteins (amyloid and tau), hippocampal volume and metabolism, and cognitive performance. We analyzed baseline CSF heme/iron proteins (multiplexed mass spectrometry-based assay), amyloid and tau (Luminex platform), baseline/longitudinal neuroimaging (MRI, FDG-PET) and cognitive outcomes in 86 cognitively normal, 135 mild-cognitive impairment and 66 Alzheimer's participants from the Alzheimer's Disease Neuroimaging Initiative-1 (ADNI-1) cohort. Multivariate regression analysis was performed to delineate differences in CSF proteins between diagnosis groups and evaluated their association to amyloid and tau, neuroimaging and cognition. A *p*-value ≤ 0.05 was considered significant. Higher hemopexin was associated with higher CSF amyloid (implying decreased brain amyloid deposition), improved hippocampal metabolism and cognitive performance. Meanwhile, hemoglobin subunits were associated with increased CSF tau (implying increased brain tau deposition). When dichotomizing individuals with mild-cognitive impairment into stable and converters to Alzheimer's disease, significantly higher baseline hemoglobin subunits were observed in the converters compared to non-converters. Heme/iron dyshomeostasis is an early and crucial event in AD pathophysiology, which warrants further investigation as a potential therapeutic target.

## Introduction

The amyloid cascade hypothesis remains the major framework for explaining the pathophysiology of Alzheimer's disease (AD) (Hardy and Higgins, 1992). It assumes a serial model of causality whereby β-amyloid (Aβ) drives tau hyperphosphorylation, resulting in neuronal death and dementia. Cerebrospinal fluid (CSF) communicates freely with brain interstitial fluid (Masters et al., 2015), thus its composition reflects brain biochemistry. CSF-based biomarkers for AD have been identified including CSF Aβ and tau–these are now used alongside cognitive and imaging biomarkers when diagnosing clinically probable AD (Dubois et al., 2014). Importantly, AD pathology can be present in individuals with mild cognitive impairment (MCI), with 10–15% of these individuals progressing to AD annually (Petersen et al., 1999). However, this group displays heterogenicity as not all convert to AD. Additionally, with the recent failure of anti-Aβ therapies, there is a need to identify alternative mechanisms of disease pathogenesis, including the contribution of iron dyshomeostasis. Indeed, we have recently provided evidence of a form of iron-dependent cell death termed ferroptosis in post-mortem AD brains (Ashraf and So, 2020; Ashraf et al., 2020b).

Abnormal iron metabolism contributes to oxidative stress and neurodegeneration in AD. While iron is essential for cellular function, it is detrimental in excess (Shah et al., 2011; Ayton et al., 2015; Ashraf et al., 2018, 2019b). Hemoglobins (Hb) are iron-containing proteins that interact with Aβ and found to be colocalized with Aβ plaques in AD brains at post-mortem (Wu et al., 2004; Chuang et al., 2012). The augmented expression of Hb subunits α and β have been demonstrated in aged human and rat brains (Blalock et al., 2003; Richter et al., 2009), and associated with cognitive deficits. The oxidation of Hb subunits enables free heme to augment redox-active iron, leading to lipid peroxidation via the Fenton reaction (Papanikolaou and Pantopoulos, 2005). Heme/iron mediated toxicity is attenuated through a scavenger protein hemopexin (HPX), which avidly binds to and regulates heme biology to ensure iron homeostasis (Tolosano and Altruda, 2002; Smith and Mcculloh, 2015).

We have recently documented increased plasma hemopexin levels to be associated with increased brain amyloid uptake as measured by ^18F^-florbetaben position emission tomography (amyloid PET), in a cohort comprising cognitively normal (CN), MCI and AD (Ashraf et al., 2020a). Moreover, increased plasma HPX was associated with a lower clinical dementia rating (CDR), suggestive of improved cognition. Our plasma analysis suggested iron dyshomeostasis is implicated in AD pathogenesis. The aim of the present study was to extend our previous findings of plasma to the CSF, albeit in a different cohort CSF being a more direct measure of the brain metabolism. We hypothesized that increased CSF HPX would be associated with lower AD pathology, improved hippocampal volume and glucose metabolism, and improved cognitive performance. Moreover, we anticipated that CSF Hbβ levels would be associated with worse outcomes.

## Materials and Methods

### ADNI Study

Participants (*n* = 287) from the Alzheimer's Disease Neuroimaging Initiative-1 (ADNI-1) study were included (http://adni.loni.usc.edu/). This group comprised 86 CN participants, 135 participants with MCI and 66 participants with AD. All the participants underwent apolipoprotein E (APOEε4) genotyping. ADNI uses serial clinical and neuropsychological assessments, imaging and CSF biomarkers to monitor progression of MCI subjects to AD. Written informed consent was obtained and approved by the institutional review board at participating centers for the use of human data in ADNI database. Data used in the preparation of this article is available on the ADNI database (adni.loni.usc.edu).

ADNI was launched in 2003 by the National Institute on Aging (NIA), the National Institute of Biomedical Imaging and Bioengineering (NIBIB), the Food and Drug Administration (FDA), private pharmaceutical companies and non-profit organizations, as a $60 million, 5-year public-private partnership. The primary goal of ADNI has been to test whether serial magnetic resonance imaging (MRI), PET, other biological markers, and clinical and neuropsychological assessment can be combined to measure the progression of MCI and early AD. Determination of sensitive and specific markers of very early AD progression aids researchers/clinicians to develop new treatments and monitor their effectiveness, and identifies suitable participants for enrolment clinical trials. The Principal Investigator of this initiative is Michael W. Weiner, MD, VA Medical Center and University of California San Francisco, with many co-investigators from a broad range of academic institutions and private corporations. Subjects have been recruited from over 50 sites across the U.S. and Canada, for up-to-date information, please see http://www.adni-info.org/.

#### Inclusion/Exclusion Criteria

Enrolled subjects in the ADNI-1 cohort were 55–90 years of age, accompanied by a study partner able to provide independent evaluation of recruited participant's functioning, and speak either English or Spanish fluently. Participants must have a Hachinski Ischaemic score ≤4, geriatric depression scale <6, visual and auditory acuity adequate for neuropsychological testing, six grades education or work history and not enrolled in other trials or studies. Individuals on specific psychoactive medications e.g., narcotic analgesics, neuroleptics, anticholinergic agents, antiparkinsonian medications, investigational drugs, benzodiazepines, antihypertensive agents with frequent central nervous system side-effects, antidepressants, within the 4 weeks prior to screening were excluded. Individuals with any serious neurological disease other than AD, any history of brain lesions or brain trauma, were also excluded.

CN subjects must have no significant impairment in their cognitive domains or impaired activities of daily living, with a mini-mental state examination (MMSE) score between 24 and 30, a CDR of 0, non-depressed, no MCI and non-demented. The age range of CN individuals was matched to that of MCI and AD subjects. MCI subjects must also have an MMSE score between 24 and 30 but have a memory complaint, experience objective memory loss measured by education adjusted scores on Wechsler Memory Scale Logical Memory II, a CDR of 0.5, although no significant impairment in other cognitive domains and preserved activities of daily living, and free of dementia. The AD cases included in the study had MMSE scores between 20 and 26, CDR of 0.5 or 1.0 and met National Institute of Neurological and Communicative Disorders and Stroke-Alzheimer's Disease and Related Disorders Association criteria for probable AD (NINDS–ADRDA).

#### Neuropsychological Assessments

All subjects underwent MMSE, Rey Auditory Verbal Learning Test (RAVLT) and Alzheimer's disease assessment scale 13 (ADAS-Cog13). MMSE measures orientation, attention, memory (immediate and delayed recall) and language. RAVLT tests episodic verbal memory by assessing an individual's ability to acquire a list of 15 words over five trials. The test comprised a short-delay recall trial presented after a distracter list, and a 30 min long delay recall trial, and finally by a yes/no recognition trial (http://www.adni-info.org). ADAS-Cog13 is a 13-item scale used for assessing learning, memory, language production and comprehension, constructional and ideational praxis, orientation, has number cancellation and delayed free recall tasks. The word recall test was administered first, and the word recognition task given at the end with other cognitive tasks given in between. The two-word memory tasks were separated so that the risk of individuals confusing words from the two tasks was minimized. Objective testing was followed by subjective clinical ratings of language ability and aptitude of the participant to remember test instructions (extended details can be found on the ADNI website: adni.loni.usc.edu/wp-content/uploads/2010/09/ADNI_GeneralProceduresManual.pdf).

#### Conversion of MCI to AD

Tracking the rate of conversion from MCI to AD is a primary outcome measure of the ADNI protocol. Site physicians review participants' data and completes diagnostic summaries. If a physician triggers a change in diagnosis from MCI to AD, the clinical monitor onsite reviews the neuropsychological assessments for that visit. The clinical monitor will resolve any issues with the site's primary investigator and instruct the diagnosis to be reversed if incorrectly reported. An ADNI clinical co-investigator subsequently reviews the data and requests the clinical monitor to resolve any scoring issues. When this review is finalized, the ADNI conversion committee is then commissioned with the task of reviewing all patient reports and a consensus on the conversion status of participant is achieved as per the NINDS-ARCDA (please refer to general procedures manual; http://adni.loni.usc.edu/). Although a neuropathological diagnosis is required to confirm diagnosis of AD, studies have shown high sensitivity and specificity using neuroimaging and neuropsychological assessments for determining MCI conversion to AD (Davatzikos et al., 2001, 2011; Fan et al., 2008; Petersen et al., 2010; Cabral et al., 2015). Of the 135 MCI subjects, 85 converted to AD (MCI-c), while the remaining 50 MCI non-converters (MCI-nc) did not in a period of 7 years, with most continuing to satisfy the criteria for MCI except for four, who became CN.

### CSF Analysis

CSF samples were obtained in the morning following overnight fasting at the baseline visit. The time from collection to freezing was ~1 h, with processing, aliquoting and storage at −80°C as per ADNI Biomarker Core Laboratory Standard Operating Procedures. CSF Aβ_1−42_, phosphorylated tau (ptau) and total tau (ttau) were measured using the Luminex platform as described previously (Jagust et al., 2009; Shaw et al., 2011). CSF APOE levels were determined using a Myriad Rules Based Medicine platform (Human Discovery MAP, v1.0). A multiplexed mass spectrometry (MS)-based assay using multiple reaction monitoring (MRM) was used to detect CSF levels of HPX (NFPSPVDAAFR), Hbα (FLASVSTVLTSK) and Hbβ (EFTPPVQAAYQK). The methods were developed by Caprion Proteomics in collaboration with the ADNI Biomarker Consortium Project team. The technology, quality control and validation of the MRM platform is fully described in the “Use of Targeted Mass Spectrometry Proteomic Strategies to Identify CSF-Based Biomarkers in Alzheimer's Disease Data Primer” on the ADNI website (ida.loni.usc.edu) and elsewhere (Percy et al., 2014; Spellman et al., 2015).

### Structural MRI Volumes

Subjects underwent structural MRI at 1.5T using a 3D sagittal volumetric magnetization prepared rapid gradient echo (MP-RAGE) sequence (Jack et al., 2010). The acquisition parameters were: repetition time, 9 ms; echo time, 4 ms; flip angle 8°, with a 256 × 256 × 170 acquisition matrix in the x-, y- and z-dimensions with a nominal voxel size of 0.94 × 0.94 × 1.2 mm^3^. MRI was performed at baseline, 6 months, 1 year, then yearly for 6 years. FreeSurfer (version 4.1.0) was used to calculate hippocampal volumes and described previously (Fischl et al., 2002, 2004). Briefly, MRI volumetric images initially underwent motion correction (Reuter et al., 2010), hybrid watershed or surface deformation removal of non-brain tissue (Segonne et al., 2004), automated Talairach transformation, and then segmentation of subcortical white and deep gray matter structures (Fischl et al., 2002). This was followed by intensity normalization (Sled et al., 1998), tessellation of gray and white matter boundary, and automated topology correction (Segonne et al., 2007). The hippocampus and amygdala have similar signal intensities, but their spatial location is quite consistent relative to one another, the amygdala is always in front of and above the hippocampus. To ensure the segmentations were anatomically plausible, the Markov random field model was used and modified to be spatially non-stationary (Fischl et al., 2002, 2004; Fischl, 2012). This involves separately modeling the probabilities of the hippocampus above and below the amygdala, resulting in accurate identification of the hippocampus. Hippocampal volume was calculated by multiplying the number of hippocampal voxels by the voxel volume.

### [^18^F] Fluorodeoxyglucose ([^18^F]FDG-PET)

[^18^F]FDG-PET scans were acquired on multiple scanners at various resolutions at 6 months, 1, 1.5, and 2 years (Jagust et al., 2010). The scans were acquired as 6 × 5-min frames, from 30 min after injection of 5 mCi of ^18^F-FDG (full details at http://www.adni-info.org/Scientists/doc/PET-Tech_Procedures_Manual_v9.5.pdf). The FDG-PET images were pre-processed according to standard ADNI procedures with frames co-registered, averaged and reoriented along the anterior-posterior commissure line and resliced to a 1.5 mm isotropic voxel space. Each PET image was spatially normalized to Montreal Neurological Institute (MNI) space and the mean hippocampal FDG uptake (normalized to pons uptake) measured (http://adni.loni.usc.edu/methods/pet-analysis/pre-processing/).

## Statistical Analysis

Statistical analysis was performed using SPSS IBM version 24.0. GraphPad Prism 8.4.2 (GraphPad Inc., San Diego, CA) was used to compute heatmaps. We used MANCOVA modeling to assess the differences in CSF proteins (HPX, Hbα, and Hbβ) across diagnostic groups, with age, sex and APOEε4 genotype included as covariates. The advantages of using MANCOVA extends the capabilities of ANCOVA by enabling assessment of multiple dependent variables simultaneously. MANCOVA require additional *p*-value correction as in the case of univariate analysis, hence the error rate equals the significance level. Moreover, MANCOVA assesses the pattern between multiple dependent variables which is not detected in ANCOVA analysis, leading to greater statistical power to locate differences between groups.

We also repeated the MANCOVA analysis (as above), but this time to assess differences across CSF biomarker negative CN, and biomarker positive MCI and AD subjects, rather than solely across diagnostic groups. Subjects were stratified according to the previously published CSF total tau/Aβ ratio threshold ratio (Ayton et al., 2015; Shaw et al., 2018): a ratio <0.27 being negative and ≥0.27, positive.

In addition, we undertook partial correlation analysis adjusted for APOEε4 genotype and disease status to understand the relationship of CSF HPX, Hbα, and Hbβ to baseline levels of CSF APOE, Aβ, ptau, ttau, neuroimaging (hippocampal volume and metabolism) and cognitive (MMSE, RAVLT and ADAS-Cog13) measures. We also examined the association of CSF proteins with longitudinal changes in neuroimaging and cognitive measures, which were computed using the following equation:

$$\begin{array}{l} \text{longitudinal change}\\ =\frac{\text{Follow}-\text{up timepoint measure }-\text{ Baseline measure}}{\text{Baseline measure}} \end{array}$$

We extended the findings from the partial correlation analysis by conducting multivariate regression modeling to confirm the associations. Since follow-up times were different between subjects, follow-up time was included as a covariate. A minimal model-based approach was utilized using the Akaike information criterion (AIC) and Bayesian information criterion (BIC) to yield the most appropriate fitted model.

MCI is known to be a heterogeneous group, with differing rates of progression to AD, these individuals were a focus of this study. We grouped MCI subjects according to their CSF biomarker status, to examine relationships between biomarker status and levels of HPX, Hbα, and Hbβ. We also compared baseline HPX, Hbα, and Hbβ levels between MCI-c and MCI-nc, but only in those MCI individuals who were CSF biomarker positive, to determine if they are altered prior to conversion from MCI to AD. Two-tailed t-tests were computed to assess the differences.

For statistical analysis, we tested data satisfied assumptions by checking for collinearity, normal distribution of residuals, maintenance of homoscedasticity and normality of error terms, using Levene's test, Box's test of equality of covariance matrices, histogram, P-P and Q-Q plots. The regression standardized predicted values were plotted against standardized regression residuals and if points were found to be equally distributed with no obvious pattern, homoscedasticity was confirmed. Aβ, ptau and ttau were log-transformed to ensure normality. Values were quoted as mean ± standard deviation (S.D.). A *p*-value ≤ 0.05 was considered significant for MANCOVA and multiple regression analysis. A false discovery rate (FDR) correction set at Q of 5% was then applied to correct for multiple comparisons for *t*-tests (corrected *p* ≤ 0.0397) and partial correlation analysis (corrected *p* ≤ 0.036).

## Results

### Demographic Details

Of our ADNI cohort, most participants were white non-Hispanic (*n* = 273) while the remaining were white Hispanic (*n* = 4), black non-Hispanic (*n* = 10) and asian non-Hispanic (*n* = 3). The age ranges for CN (75.70 ± 5.54 years), MCI (74.69 ± 7.35 years) and AD (74.98 ± 7.57 years) were similar. The CN group had 49% females, MCI had 33% while AD, 29%. The years of education was similar in CN (15.64 ± 2.97), MCI (16 ± 2.97) and AD (15.11 ± 2.96). APOEε4-positivity was observed in 24% of CN, 53% of MCI and 71% of AD.

### Baseline Characteristics

The baseline levels of CSF HPX (*p* = 0.836), Hbα (*p* = 0.814), and Hbβ (*p* = 0.997) were similar in CN, MCI and AD (Table 1). Likewise, stratification of subjects according to their CSF biomarker status, i.e., including only CSF biomarker-negative CN and CSF biomarker-positive MCI and AD individuals), did not result in significant changes in CSF HPX (*F* = 0.437, *p* = 0.646), Hbα (*F* = 0.230, *p* = 0.795) or Hbβ (*F* = 0.074, *p* = 0.929) between diagnostic groups. Expectedly, CSF Aβ levels (*p* = 2.369 × 10^−7^) were significantly decreased with advancing disease (CN > MCI > AD). CSF ptau (*p* = 6.051 × 10^−8^) and ttau (*p* = 1.492 × 10^−7^) were significantly increased in MCI and AD. Meanwhile, hippocampal atrophy (*p* = 6.587 × 10^−16^) and glucose hypometabolism (*p* = 1.438 × 10^−17^) were pronounced in MCI, with further worsening observed in AD. The cognitive performance measured by MMSE (*p* = 3.255 × 10^−48^), RAVLT (*p* = 7.620 × 10^−34^) and ADAS-Cog13 (*p* = 3.231 × 10^−43^) was significantly impaired with advancement in disease.

**Table 1:** Baseline levels of CSF proteins, neuroimaging and cognitive measures stratified by diagnosis.

	**CN**	**MCI**	**AD**	**F**	***p*-value**
N	86	135	66	–	–
HPX (a.u.)	29.242, 0.715	29.291, 0.745	29.368, 0.795	0.180	0.836
Hbα (a.u.)	15.426, 2.959	15.212, 2.863	15.660, 2.983	0.206	0.814
Hbβ (a.u.)	12.484, 4.741	12.377, 4.422	12.722, 4.970	0.003	0.997
Aβ (pg/ml)	209.256, 53.386	161.024, 49.963	141.326, 35.729	16.148	**2.369** **×** **10**^**−7**^
ptau (pg/ml)	24.117, 11.968	35.252, 15.127	41.955, 20.601	17.683	**6.051** **×** **10**^**−8**^
ttau (pg/ml)	70.333, 27.638	102.993, 51.677	126.172, 60.691	16.666	**1.492** **×** **10**^**−7**^
HipVol (mm^3^)	7159, 845	6212, 1075	5681, 1099	39.879	**6.587** **×** **10**^**−16**^
HipFDG	1.310, 1.290	1.200, 0.129	1.070, 0.135	44.900	**1.438** **×** **10**^**−17**^
MMSE	29.060, 1.033	26.920, 1.737	23.520, 1.850	168.169	**3.255** **×** **10**^**−48**^
RAVLT	43.240, 8.514	29.940, 8.452	22.580, 7.569	102.377	**7.620** **×** **10**^**−34**^
ADAS-Cog13	9.337, 4.215	19.013, 6.106	29.288, 8.291	143.448	**3.231** **×** **10**^**−43**^

*Data is presented mean ± standard deviation. Age, sex, APOEε4 genotype adjusted MANCOVA. For the analysis, Aβ, ptau and ttau were log-transformed to ensure normality. A p-value < 0.05 was considered significant; significant values are in Bold. HPX, Hemopexin; Hbα, Hemoglobin α; Hbβ, Hemoglobin β; Aβ, β-amyloid; ptau, phosphorylated tau; ttau, total tau; HipVol, hippocampal volume; HipFDG, hippocampal FDG; MMSE, mini-mental state examination; RAVLT, Rey Auditory Verbal Learning Test; ADAS-Cog13, The Alzheimer's Disease Assessment Scale–Cognitive Subscale. HPX, Hbα, and Hbβ was measured in arbitrary units (a.u.) on a natural log scale*.

### Association of CSF Heme Proteins to AD Pathology, Neuroimaging and Cognitive Measures

**Figure 1:**
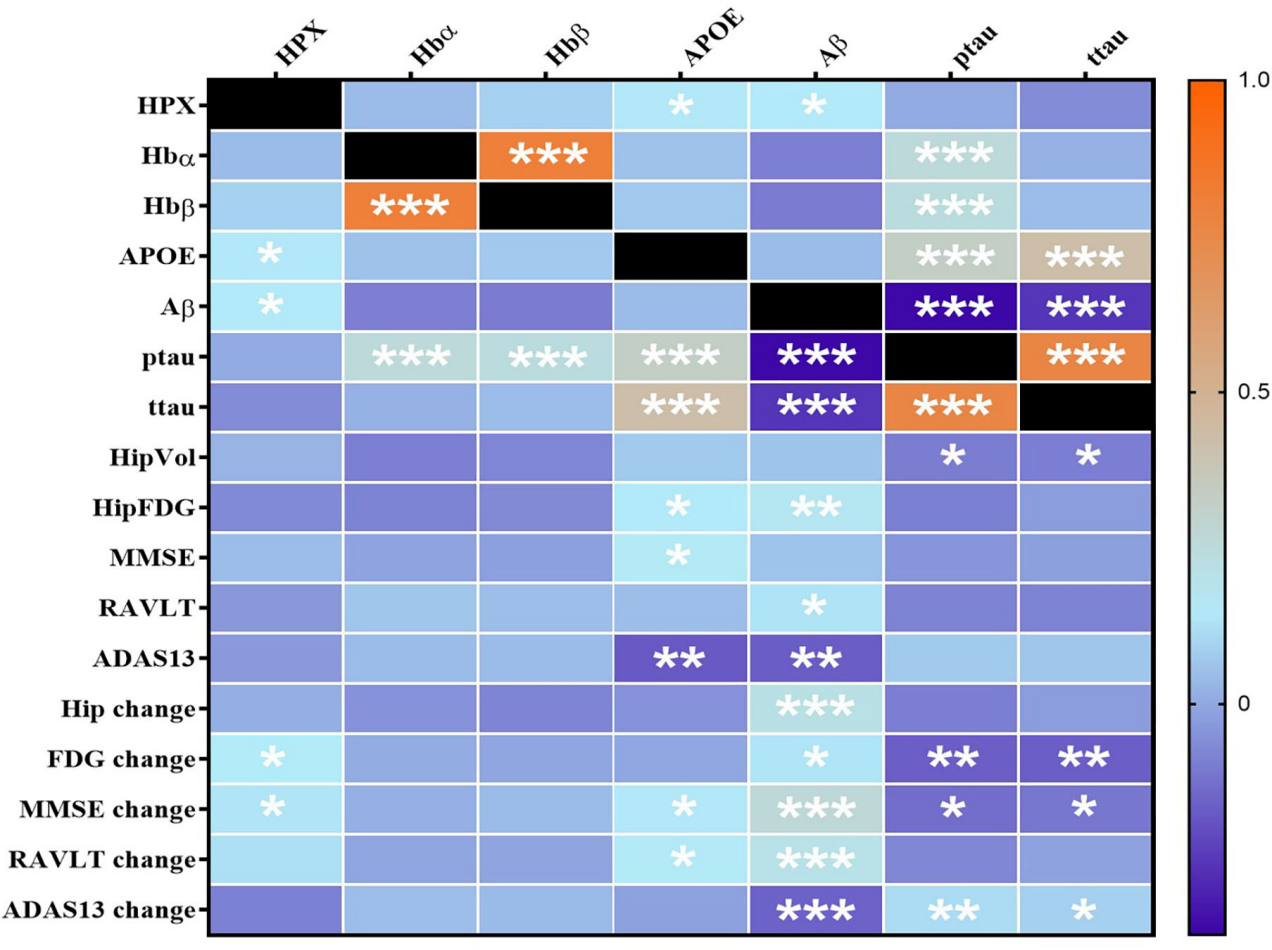
Heatmap of partial correlations between CSF proteins adjusted for APOEε4 genotype and diagnosis. The scale-bar represents the correlation coefficient (r). Aβ, ptau and ttau were log-transformed to ensure normality. An FDR-corrected p-value ≤ 0.036 was considered significant. *p ≤ 0.036; **p ≤ 0.005, ***p ≤ 0.0005. HPX, Hemopexin; Hbα, Hemoglobin α; Hbβ, Hemoglobin β; APOE, Apolipoprotein E; Aβ, β-amyloid; ttau, total tau; ptau, phosphorylated tau; HipVol, hippocampal volume; HipFDG, hippocampal ^18^F-fluorodeoxyglucose; MMSE, mini-mental state examination; RAVLT, Rey Auditory Verbal Learning Test; ADAS-Cog13, The Alzheimer's Disease Assessment Scale–Cognitive Subscale.

**Figure 2:**
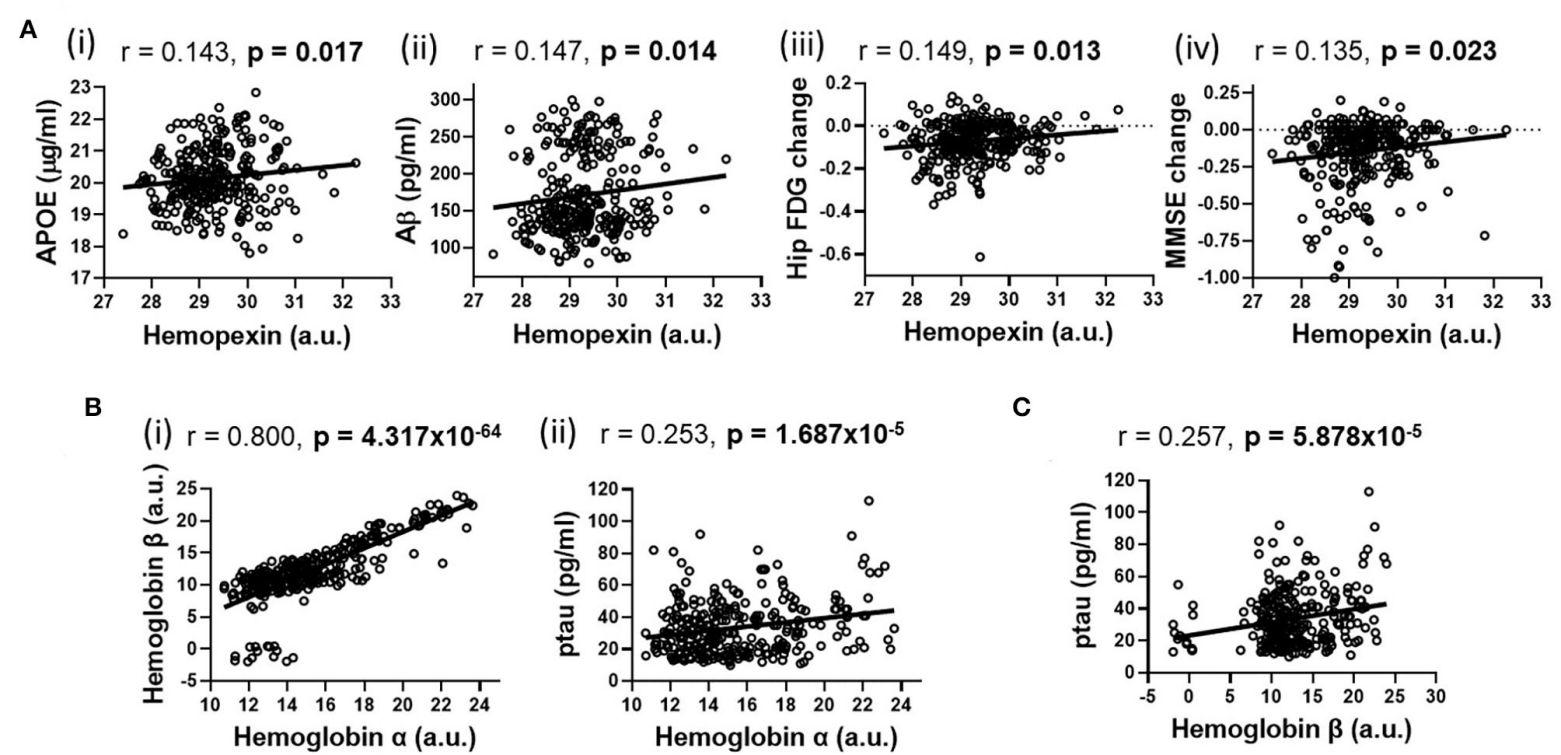
Partial correlations adjusted for APOEε4 genotype and diagnosis illustrating significant associations (shown in heatmap in Figure 1) between **(A)** hemopexin with (i) APOE, (ii) β-amyloid (Aβ), (iii) longitudinal changes in hippocampal glucose (FDG) metabolism and (iv) longitudinal mini-mental state examination (MMSE) change; **(B)** Hemoglobin α with (i) Hemoglobin β and (ii) phosphorylated tau (ptau); **(C)** Hemoglobin β with ptau. An FDR-corrected *p*-value ≤ 0.036 was considered significant.

Using partial correlation analysis adjusted for diagnosis and APOEε4 genotype (Figures 1, 2), higher levels of CSF HPX were significantly associated with increased CSF APOE (*r* = 0.143, *p* = 0.017) and CSF Aβ (*r* = 0.147, *p* = 0.014) levels; and improved longitudinal changes in glucose metabolism (*r* = 0.149, *p* = 0.013), MMSE (*r* = 0.135, *p* = 0.023) and RAVLT (*r* = 0.123, *p* = 0.038, but did not remain significant after FDR correction) scores. A positive correlation was observed between CSF Hbα and Hbβ levels (*r* = 0.800, *p* = 4.317 × 10^−64^). High levels of CSF Hbβ were related to lower levels of CSF Aβ but at a non-significant level (*r* = −0.103, *p* = 0.085). Higher levels of CSF Hbα (*r* = 0.253, *p* = 1.687 × 10^−5^) and Hbβ (*r* = 0.257, *p* = 5.878 × 10^−5^) were significantly associated with increased levels of CSF ptau. CSF HPX (*r* = −0.065, *p* = 0.276), Hbα (*r* = 0.021, *p* = 0.728) and Hbβ (*r* = 0.046, *p* = 0.446) levels were not significantly associated with CSF ttau.

### Multivariate Modeling Confirms Associations

Initially, we devised our model using CSF HPX, Hbα, and Hbβ as independent variables, with baseline CSF APOE, Aβ, ptau and longitudinal changes in hippocampal metabolism, MMSE and RAVLT scores as dependent variables. The ttau levels, baseline scores of neuroimaging and cognitive performance were not included as they showed no association with either CSF HPX, Hbα or Hbβ based on partial correlation analysis. Further, as CSF Hbα and Hbβ showed significant positive correlation, multicollinearity was detected, which renders the relative strength of predictor variables and their interaction effects unreliable. To ensure absence of collinearity, we selected CSF Hbβ and excluded CSF Hbα as the former was a better fit for the model. In the process, the model fit was improved from an AIC of 492.719 and BIC of 602.504 to 471.186 and 548.035, respectively. To ensure the central theorem hypothesis (and multivariate normality) was maintained, we had to remove longitudinal change in the MMSE variable. The finalized model included CSF HPX and Hbβ as predictors to the model which included CSF APOE, Aβ, ptau, longitudinal changes in hippocampal metabolism and RAVLT as dependent variables. The finalized model had an improved AIC of 285.768 and BIC of 351.638, confirming the achievement of a best-fitted model.

**Figure 3:**
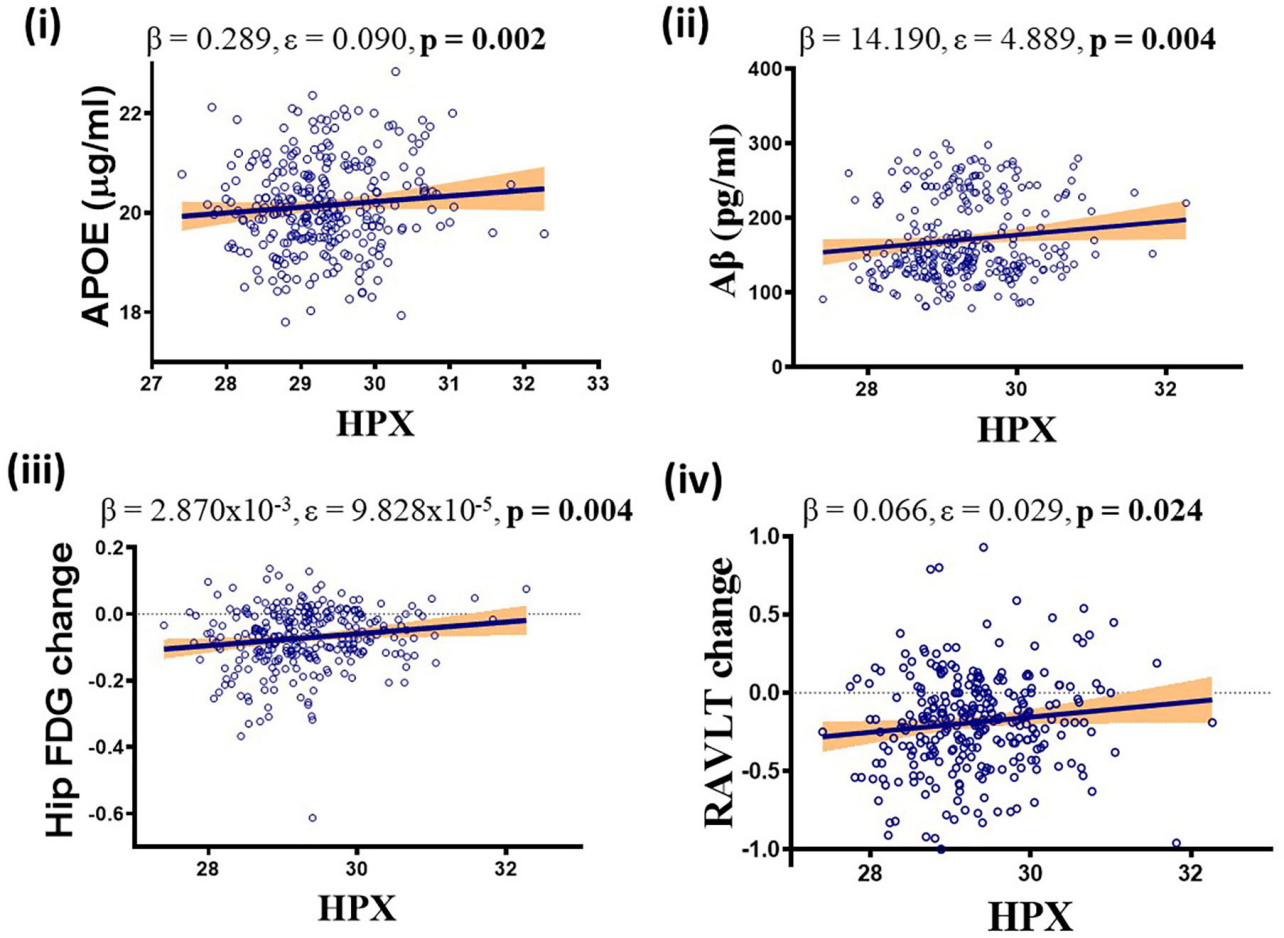
APOEε4, diagnosis and follow-up time adjusted multivariate analysis demonstrating significant positive association between CSF hemopexin (HPX) and (i) apolipoprotein E (APOE), (ii) β-amyloid (Aβ), (iii) hippocampal glucose (FDG) metabolism and (iv) Rey Auditory Verbal Learning Test (RAVLT). Unstandardized coefficient (β), standard error (ε) and *p*-values are stated along with correlation graphs. A *p* ≤ 0.05 was considered significant.

Higher levels of CSF HPX were associated with increased CSF APOE (β = 0.289, ε = 0.090, *p* = 0.002) and CSF Aβ (β = 14.190, ε = 4.889, *p* = 0.004) levels, improved longitudinal hippocampal glucose metabolism (β = 2.870 × 10^−3^, ε = 9.828 × 10^−5^, p = 0.004) and RAVLT performance (β = 0.066, ε = 0.029, *p* = 0.024; Figure 3). No correlation was observed between CSF HPX and ptau levels (β = −0.924, ε = 1.635, *p* = 0.573). Meanwhile, higher levels of CSF Hbβ were associated with deteriorated longitudinal hippocampal glucose metabolism (β = −3.885 × 10^−5^, ε = 1.481 × 10^−5^, *p* = 0.010) and non-significantly decreased CSF Aβ (β = −0.768, ε = 0.737, *p* = 0.299), and significantly increased ptau (β = 0.857, ε = 0.246, *p* = 0.001) levels.

### Relationships Between Baseline CSF HPX, Hbα and Hbβ With MCI Conversion to AD

**Figure 4:**
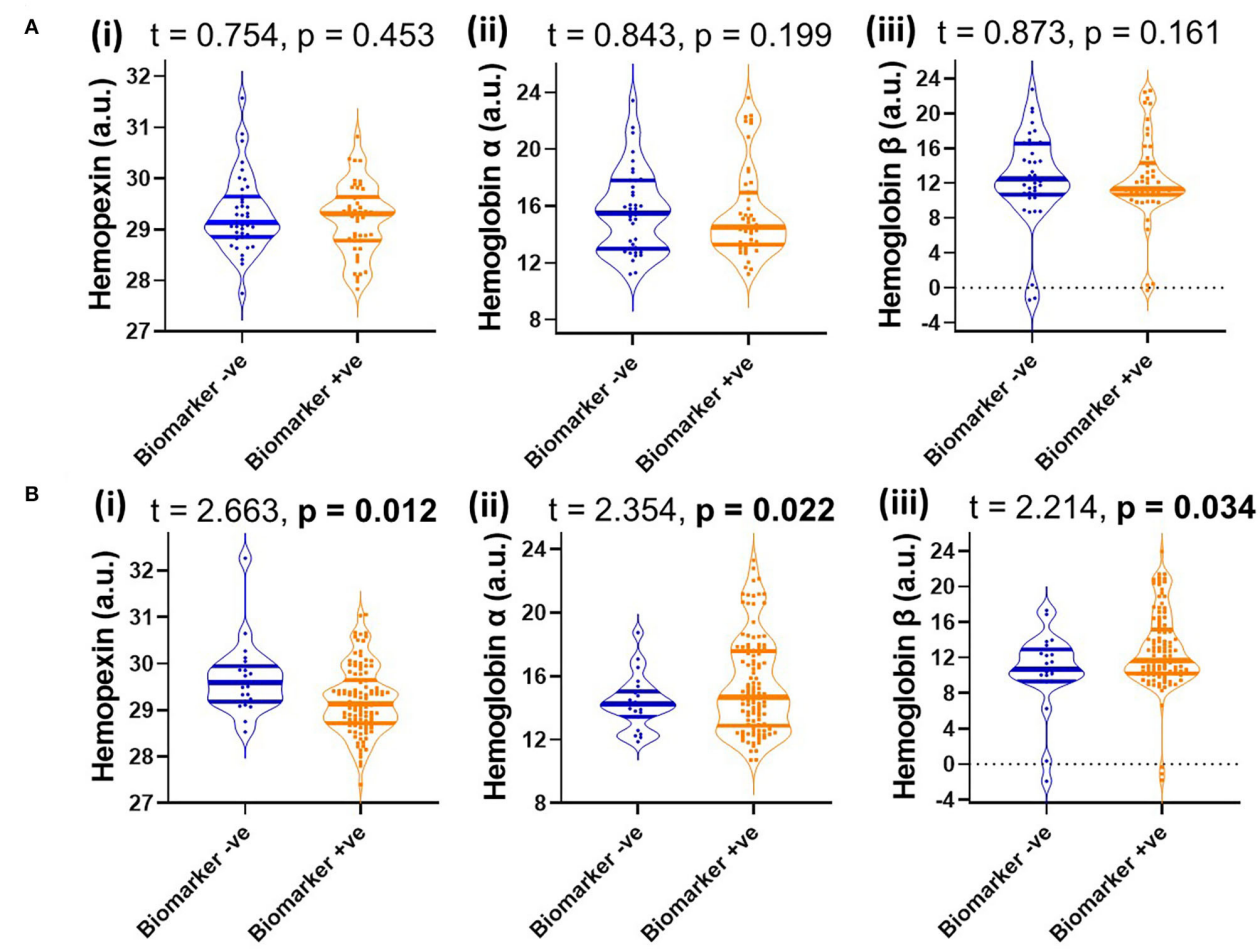
Violin plots showing CSF levels of (i) hemopexin, (ii) hemoglobin subunits α and (iii) hemoglobin subunits β in **(A)** cognitively normal **(B)** and mild-cognitive impaired individuals, stratified by biomarker status based on a threshold of total tau/Aβ (<0.27, depicts biomarker-negative and ≥0.27, biomarker-positive). Two-tailed t-testing was used to assess differences with values reported as median and interquartile range as well as t and *p*-values. An FDR corrected *p*-value ≤ 0.0397 was considered significant. Hemopexin and hemoglobin subunits were measured in arbitrary units (a.u.) on a natural log scale.

**Figure 5:**
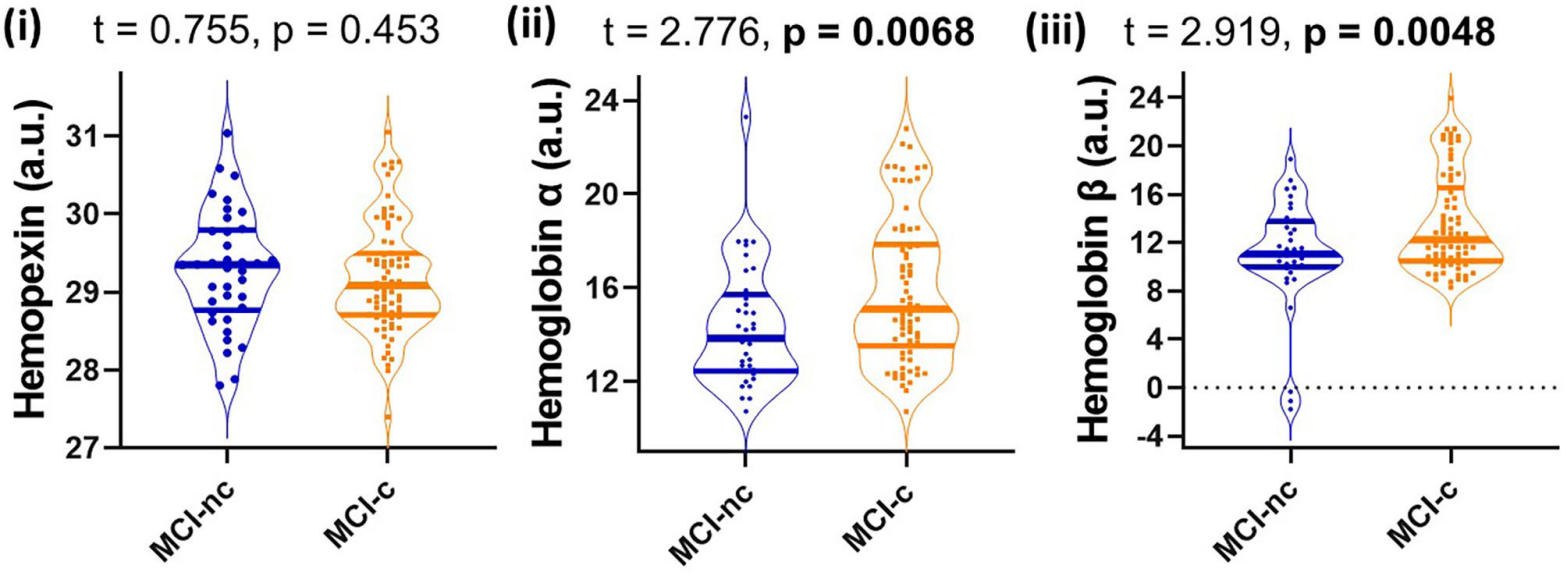
Violin plots showing CSF levels of hemopexin and hemoglobin subunits α and β in individuals with mild-cognitive impairment (MCI-nc) remaining stable and those converting (MCI-c) to Alzheimer's disease. Only those MCI subjects that are CSF biomarker-positive were included in the analysis. The biomarker status was based on a threshold of total tau/Aβ (<0.27 is biomarker-negative and ≥0.27 is biomarker-positive). Two-tailed t-testing was used to assess differences with values reported as median and interquartile range as well as t and *p*-values. An FDR corrected *p*-value ≤ 0.0397 was considered significant. Hemopexin and hemoglobin subunits were measured in arbitrary signal intensity units on a natural log scale.

Since MCI represents a heterogenous group at heightened risk of converting to AD, we assessed baseline levels of CSF HPX, Hbα and Hbβ and determined their relationship with disease progression. Baseline CSF HPX levels were significantly lower in MCI biomarker-positive individuals compared to MCI biomarker-negative individuals (*p* = 0.012). However, CSF Hbα and Hbβ were higher in biomarker-positive MCI individuals compared to those that were MCI that was biomarker-negative (*p* = 0.022 and 0.0034, respectively; Figure 4). The significant changes observed in MCI were not evident in the CN group (Figure 4). When evaluating conversion status only in those MCI subjects that were biomarker-positive, baseline levels of Hbα and Hbβ were significantly higher in MCI-c compared to MCI-nc (*p* = 0.0068 and 0.0048, respectively; Figure 5), while HPX levels were similar (*p* = 0.453).

## Discussion

Consistent with our hypothesis, we demonstrate higher levels of CSF HPX were associated with improved: (1) CSF APOE, (2) CSF Aβ levels, (3) hippocampal glucose metabolism and (4) cognitive performance. Moreover, CSF Hb subunits were significantly higher in MCI-c compared to MCI-nc and were associated with decreased CSF Aβ and increased CSF ptau. The present CSF study extends our recent plasma study (Ashraf et al., 2020a), and solidifies evidence for the involvement of heme/iron dyshomeostasis in the pathogenesis of AD.

Since iron is required for Hb oxygen transport, alterations in CSF levels of Hbα and Hbβ chains support the hypothesis of disrupted iron homeostasis in AD (Altinoz et al., 2019). We observed higher CSF Hbα and Hbβ levels in MCI-c compared to MCI-nc while no significant differences were obtained between CN, MCI, and AD according to syndrome diagnosis–with or without further stratification by CSF biomarker status. Apparently, high Hb subunit levels increase the risk of AD pathogenesis. We suggest elevated CSF Hb subunits are due to leakage from damaged neurons (Richter et al., 2009) in the more toxic milieu of the MCI-c brain. Note, a stringent methodology was employed to ensure removal of plasma proteins from CSF samples and avoid false results (Spellman et al., 2015).

**Figure 6:**
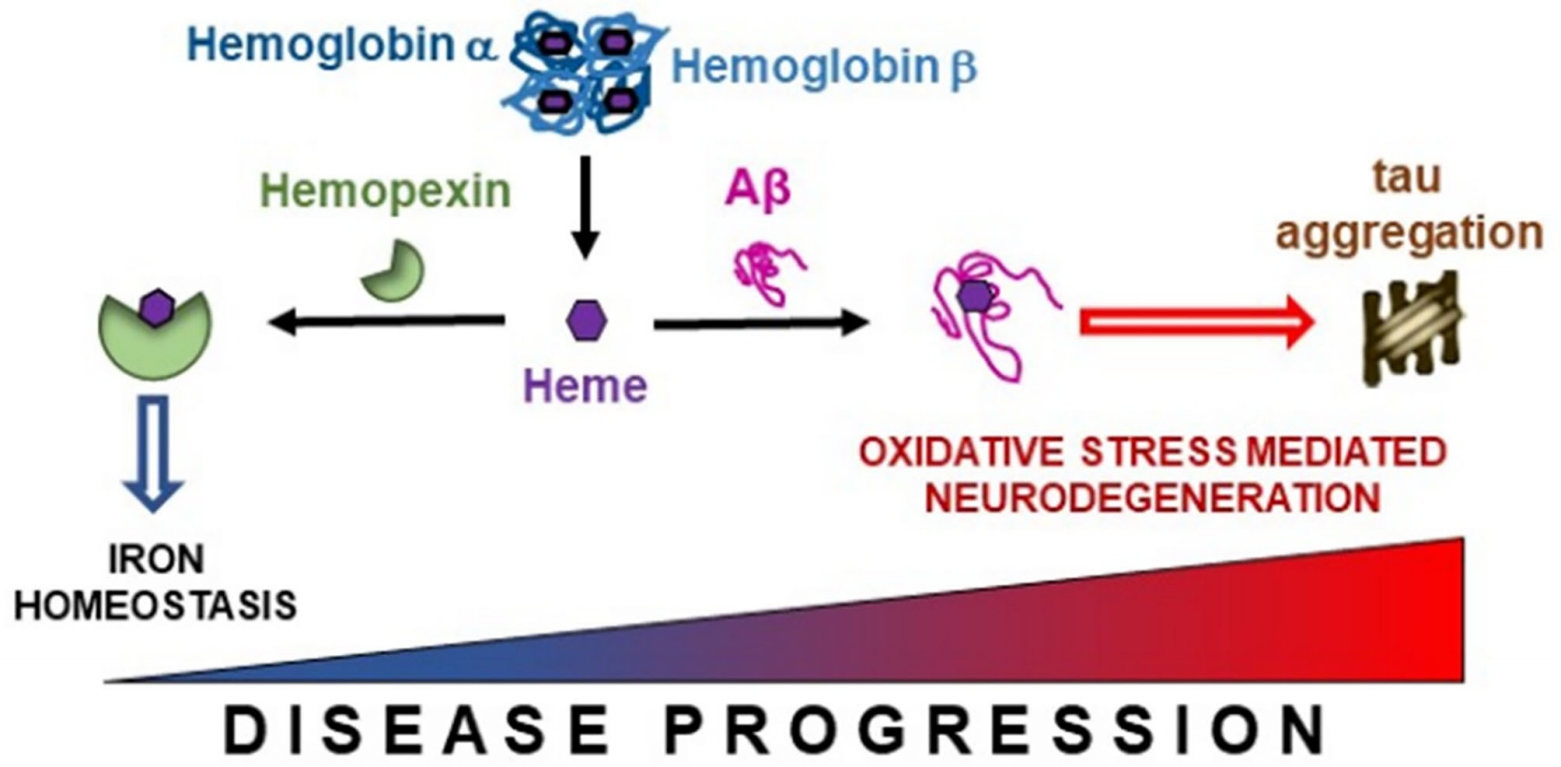
Free heme liberated from hemoglobin subunits α and β is scavenged by hemopexin. However, in the presence of accruing Alzheimer's pathology, this binding between hemopexin and heme may become disrupted enabling free heme to interact with β-amyloid (Aβ), thus promoting iron dyshomeostasis. This may lead to tau aggregation and oxidative stress mediated neurodegeneration in Alzheimer's disease.

Hb breakdown leads to the release of free heme, which is prevented from generating free radicals by being bound to HPX (Hvidberg et al., 2005; Hahl et al., 2013, 2017). HPX appears to confer neuroprotection as we demonstrated higher levels of HPX are associated with improved hippocampal metabolism and maintained cognitive performance. The heme-HPX complex is internalized by cells and then detoxified through induction of heme-oxygenase 1. Heme-oxygenase 1 degrades heme into iron, which is safely scavenged by ferritin. The remaining heme porphyrin ring is degraded to produce the anti-oxidants, biliverdin and carbon monoxide (Eskew et al., 1999; Sung et al., 2000; Vanacore et al., 2000). Combined, such metabolism maintains neuronal iron homeostasis and provide neuroprotection. Previously, Aβ has been shown to bind to heme (Atamna and Boyle, 2006), our finding of decreased CSF Aβ and so increased parenchyma Aβ deposition (Grimmer et al., 2009) is associated with lower levels of CSF HPX suggests that opportunistic Aβ as opposed to HPX, forms complexes with heme in the brain parenchyma (Figure 6). This precludes detoxification via HPX-heme complexes promoting peroxidation (Pramanik and Dey, 2011; Lu et al., 2014; Flemmig et al., 2018). The peroxidase activity of Aβ-heme complexes have been shown to induce peptide dimer formation, which in turn enhance Aβ fibrillization (Al-Hilaly et al., 2013). This proposition is favored by our concomitant finding of decreased CSF Aβ (increased brain amyloid deposition) is associated with increased CSF Hbβ (with Hbα excluded from analysis due to collinearity with Hbβ). Additionally, formation of the Aβ-heme complex can decrease heme bioavailability resulting in functional heme deficiency (Atamna and Boyle, 2006). Moreover, the positive association between Hb subunits and ptau we observed may reflect excessive build-up of Hb-derived heme that aggravates tau-derived N-terminal free-amine (R1_T_) aggregation, prolonging peroxidation and perpetuating neuronal oxidative stress (Pirota et al., 2016).

Since AD is a complex disorder characterized by multifaceted disease processes, the effect sizes we obtained in this study were small, albeit significant. This suggests that HPX and Hb subunits may not be the most appropriate biomarkers to differentiate between diagnostic groups. However, this study highlights their importance in disease pathogenesis, especially as Hb subunits are elevated in AD-affected brain regions e.g., inferior temporal gyrus (Wu et al., 2004).

Low baseline CSF HPX levels were observed in MCI individuals who are CSF biomarker-positive, concomitant with elevated levels of CSF Hbα and Hbβ subunits, which would contribute to inefficient HPX scavenging of heme in AD pathogenesis. This is consistent with the association of low CSF HPX levels with low CSF Aβ, high CSF ptau, increased glucose hypometabolism and cognitive decline, i.e., disease progression (although not MCI conversion to AD). In our previous study (Ashraf et al., 2020a), plasma HPX was associated with brain amyloid deposition, albeit from a different cohort. HPX is an abundant plasma protein that is also expressed by both neurons and glia (Morris et al., 1993), with the majority of CSF HPX produced intrathecally in normal human subjects (Garland et al., 2016). While matched plasma and CSF were not available from the present cohort, considering our previous plasma (Ashraf et al., 2020a) and current CSF HPX findings, we suggest the decreased CSF HPX associated with decreased CSF Aβ and increased ptau, and disease progression (albeit not MCI conversion to AD), may result from: abnormally greater HPX export from the CSF to the plasma compartment; decreased neuroglial HPX synthesis; and/or increased brain parenchymal low density lipoprotein receptor-related protein 1 (LRP1)-mediated scavenging (Hvidberg et al., 2005). Interestingly, LRP1 is rather a promiscuous receptor and APOE and Aβ are all ligands (Ashraf et al., 2019a) as well as HPX (Hvidberg et al., 2005). We have previously suggested that low CSF melanotransferrin (MTf) levels in MCI-c compared to MCI-nc (diagnosed according to clinical syndrome) may have been due to attenuated competitive clearance of Aβ due to its low levels in the CSF (Ashraf et al., 2019a). We propose a similar situation may occur here, with low CSF HPX levels arising from increased export from the CSF to the blood at the choroid plexus, due to lesser competition from Aβ and APOE, whose levels are low in the CSF when HPX is low, via their common receptor, LRP1. Evidently, not only does the relationship between CSF and plasma protein levels needs to be elucidated with matched samples from the same subjects, but also the relationship between brain parenchymal and CSF protein.

It is worth noting that the correlations in this study are weak but significant. Since correlational analysis does not equate to causation, further studies need to be undertaken, including measurement of HPX and Hb subunits in a separate set of samples, preferably with matched CSF and plasma samples, to assess the reproducibility of the associations observed in the ADNI dataset. Further investigations are required to determine a possible causal relationship between HPX and AD pathogenesis. Importantly, studies should temporally evaluate CSF HPX and Hb subunits in prodromal AD subjects to understand how the levels of proteins fluctuate with disease progression. Since we did not assess the functional status of HPX, it is possible that there is loss of the scavenging capabilities of HPX due to oxidative modification (Hahl et al., 2017) in AD. Additional studies will help to assess whether replenishing HPX function through resurrection of normal heme and iron biology would be a useful therapeutic approach to ameliorate oxidative stress-mediated neurodegeneration.

We did not find significant alterations in CSF protein levels between CN, MCI and AD. This could be attributed to local regional variability in their levels, particularly in disease-affected brain areas that may not lead to changes in CSF proteins levels, since CSF levels results from the summed metabolism of the whole brain.

Most importantly, iron dyshomeostasis is an attractive proposition to investigate, as it is not specific to AD, but also observed in other neurodegenerative diseases. The close ties of iron to the pathological proteins (Robinson et al., 2018), Aβ, tau, α-synuclein and TDP43, means that successful therapeutic targets involving iron biology would be translatable to several neurodegenerative diseases.

In conclusion, heme/iron dyshomeostasis is an early and crucial event in AD pathophysiology, which warrants thorough investigation as a potential therapeutic target.

## Data Availability Statement

Publicly available datasets were analyzed in this study. This data can be found here: https://ida.loni.usc.edu/pages/access/studyData.jsp.

## Ethics Statement

The studies involving human participants were reviewed and approved by As per ADNI protocols, all procedures performed in studies involving human participants were in accordance with the ethical standards of the institutional and/or national research committee and with the 1964 Helsinki declaration and its later amendments or comparable ethical standards. More details can be found at adni.loni.usc.edu. The patients/participants provided their written informed consent to participate in this study.

## Author Contributions

AA contributed to the study concept, design and carried out the data analysis, and drafted the manuscript. MD helped revise the manuscript. P-WS contributed to data interpretation and revised the manuscript. All authors read and approved the final manuscript.

## Conflict of Interest

The authors declare that the research was conducted in the absence of any commercial or financial relationships that could be construed as a potential conflict of interest.
